# Assessing the Impact of Hematological Changes in Pregnancy on Maternal and Fetal Death: A Narrative Review

**DOI:** 10.7759/cureus.66982

**Published:** 2024-08-16

**Authors:** Mayuri N Paradkar, Idalia Mejia, Rasha Abraheem, Esaúl Marroquín León, Afreen Firdous, Maria Jimena Barroso, Dexith Kumar Sampathkumar, Zoya Morani

**Affiliations:** 1 Department of Geriatrics and General Medicine, Blackpool Victoria Hospital, Blackpool Teaching Hospitals and NHS Foundation Trust, Blackpool, GBR; 2 Department of Medicine, Universidad Católica de Honduras, San Pedro Sula, HND; 3 Department of Obstetrics and Gynecology, Benghazi Medical Hospital, Benghazi Medical University, Benghazi, LBY; 4 Department of Medicine, Facultad de Medicina de la UW, Santa Fe, MEX; 5 Department of Medicine and Surgery, Shadan Institute of Medical Sciences, Hyderabad, IND; 6 Department of Internal Medicine, Universidad Anáhuac México, Mexico City, MEX; 7 Department of Medicine, University of Perpetual Help System, Las Pinas, PHL; 8 Department of Family Medicine, Ascension All Saints - Family Health Center, Milwaukee, USA

**Keywords:** thrombophilia in pregnancy, peripartum physiological changes, twin gestation, fetal mortality, maternal mortality, anemia in pregnancy, hematological changes in pregnancy

## Abstract

Hematological changes during pregnancy encompass a wide range of alterations in blood composition and function, including variations in hemoglobin levels, red blood cell count, and coagulation factors. These changes can be physiological or pathological and may significantly impact maternal and fetal health outcomes. This narrative review examines the relationship between various hematological changes and disorders during pregnancy and their effects on maternal and fetal mortality and morbidity. We explore conditions such as anemia, sickle cell disease, thrombophilia, and blood-borne infections like malaria, as well as the impact of multiple pregnancies on hematological parameters. The review also discusses the effects of COVID-19 on maternal hematology. Key findings include the high prevalence of adverse perinatal outcomes associated with these conditions, including early miscarriages, preterm birth, low birth weight, intrauterine growth restriction, and increased risk of maternal complications. The importance of early screening, diagnosis, and appropriate management of hematological disorders during pregnancy is emphasized. This review highlights the need for a multidisciplinary approach to managing pregnant women with hematological changes to optimize maternal and fetal outcomes.

## Introduction and background

During pregnancy, the maternal body undergoes remarkable changes to accommodate the growing fetus. One such change is an increase in plasma volume by an average of 1,250 mL, to a higher extent than the increase in red blood cell volume, averaging 250 mL. This change causes physiological hemodilution, leading to low blood hemoglobin concentration. As plasma volume rises, there is a reduction in hemoglobin concentration which can lead to anemia and other health complications for both the mother and the developing fetus. Therefore, it is important to monitor the iron intake of pregnant women [1]. Fetal hemoglobin which is also known as HbF is a distinctive type of hemoglobin and it is important as it binds oxygen more powerfully than adult hemoglobin, facilitating oxygen transfer from mother to fetus prenatally. Fetal hemoglobin is the vital hemoglobin in fetal red blood cells during pregnancy, accounting for 60%-80% of total hemoglobin in a full-term newborn.

Nutrition during pregnancy is a global public health issue. Poor nutrition and a lack of essential macro and micronutrients in mothers during pregnancy have been linked to higher rates of illness and death in mothers [2]. Additionally, women with sickle cell disease (SCD) and their newborns face greater health risks during pregnancy. Sickle cell anemia is caused by homozygous mutation (hemoglobin S) and is exhibited as chronic anemia with painful episodes. The most important defect that triggers these events is impaired microcirculation caused by to sickling of erythrocytes. Maternal mortality is two to three times greater than healthy pregnant women. The risk of pre-eclampsia, thromboembolism, acute pain crisis, and sepsis are also elevated. Moreover, babies risk stunted growth, stillbirth, preterm delivery, and death during childbirth [3,4]. Twin pregnancies tend to have elevated risks of twin-to-twin transfusion syndrome and twin anemia polycythemia sequence. Twin-to-twin transfusion syndrome occurs when there is an alteration of amniotic fluid sacs between the donor and the recipient fetus [5]. Recently found data also suggested that pregnant patients' infected with SARS-CoV-2 disease can present with laboratory changes during pregnancy involving changes in C-reactive protein, lymphopenia, leukocytosis, and thrombocytopenia [6]. Thromboembolic and hemostatic outcomes are increased in pregnant women affected by COVID-19 [7,8]. In endemic areas, malaria infection during pregnancy presents unique and challenging health concerns due to the complexity of maternal physiology, and the interaction between the maternal immune system, placental biology, and plasmodium parasite pathogenicity. The presence of malaria gives rise to a spectrum of complications for the mother and the developing fetus, such as anemia, miscarriage, stillbirth, low birth weight, preterm delivery, and even maternal death [9]. Blood disorders like thrombophilia have been linked to a range of significant complications for the mother and the fetus during pregnancy, such as recurrent pregnancy loss, late miscarriages, stillbirth, intrauterine growth restriction, preeclampsia, and HELLP syndrome (hemolysis, elevated liver enzymes, low platelet counts) [10].

Nearly all pregnancies are characterized by anemia with a Hb concentration not below 10 g/dL during term. Though, it is crucial to consider that in the majority of cases, this is simply a physiological process, rather than a deficiency state or underlying hematologic disorder. Throughout pregnancy, various physiological changes also occur which cause quantitative drops in hemoglobin levels. Pregnant women with imbalanced diets and low socio-economic backgrounds who fail to take prenatal supplements are commonly affected with iron deficiency and megaloblastic anemia. Although iron deficiency anemia (IDA) is a frequent cause of maternal death, sickle-cell anemia, thalassemia, folate and B12 deficiencies, hookworm infection, schistosomiasis, HIV infection, and postpartum depression are other probable causes. It is well known that anemia can cause adverse maternal, fetal, and neonatal outcomes [11].

The concentration of hemoglobin in the blood falls from an average of 12.5-13.0 g/dL to 11.0-11.5 g/dL if iron supplementation is not taken [12]. Iron supplementation plays an important role in ensuring the health of pregnant women. In many developing countries, iron supplementation programs aim to provide access to iron-rich supplements for pregnant women which can help prevent IDA [13]. Adequate intake of supplements such as folic acid and iron by expectant mothers plays a crucial role in normal fetal growth and development [14]. The majority of iron transfer from the expectant mother to the fetus is done in the third trimester which is crucial for the growth and development of the baby, so it is important to ensure that the expectant mother is consuming iron-rich foods to curb the adverse effects of IDA. Intake of vitamin C enhances the absorption of iron. The purpose of this study is to examine the effects of hemoglobin on the health of both the mother and the fetus. The study also focuses on the relationship between the severity of anemia during pregnancy and the likelihood of experiencing adverse outcomes by both the mother and the fetus. It also aimed to highlight the significance of iron intake in improving hemoglobin levels to reduce the associated risks.

## Review

Effects of anemia on maternal and fetal outcomes

In pregnancy, anemia is a significant challenge worldwide. It compromises the health outcomes of both the fetus and the mother. Recent studies reveal some important insights into the impact of anemia on pregnancy as well as the methods for preventing and treating it. This discussion intends to examine these findings and their consequences. The classic clinical diagnosis includes generalized weakness, lethargy, irritability, and low tolerance to work; however, the course of this condition may be asymptomatic, and, rarely, a severe case may present with congestive heart failure, or ankle edema, i.e., severe signs. During pregnancy, anemia is diagnosed when hemoglobin is less than 11 g/dL in patients living at sea level [15].

Iron deficiency anemia

IDA is a common occurrence during pregnancy, mostly owing to many risk factors such as advanced gestational age, extreme maternal age (teenage or beyond 35 years), racial minority status, a high number of prior pregnancies, short intervals between pregnancies, and inadequate iron consumption [16]. The relationship between maternal hemoglobin levels during labor and delivery and the method of delivery and short-term newborn outcomes has been investigated according to which decreased hemoglobin levels during labor raise the likelihood of needing a cesarean section; however, elevated maternal hemoglobin levels are linked to neonatal death. Both low and high maternal hemoglobin levels are linked to negative outcomes; hence, ideal hemoglobin levels need to be maintained during pregnancy [17]. 

Moreover, IDA can lead to neurocognitive impairments in newborns [18]. The unborn child is susceptible to changes in brain metabolism, neurotransmission, myelination, and epigenetics that can have implications that persist into adulthood. Additionally, the fetal cardiovascular system may experience diminished circulation, delayed cardiovascular development, and an oversized heart, potentially triggering autism [19]. Maternal IDA is linked to a higher likelihood of postpartum depression, worse quality of life, severe postpartum hemorrhage (PPH), increased hospitalization to maternal intensive care units, hysterectomy, maternal shock, heart failure, and maternal mortality [20,21]. These results highlight the need to monitor maternal hemoglobin levels to reduce negative delivery outcomes, emphasizing the importance of screening for hemoglobin and hematocrit levels throughout the entire pregnancy to detect and intervene early [16,17]. Interventions targeting anemia in pregnancy, such as improved prenatal care, iron supplements, and iron-fortified diets, show potential in decreasing maternal death rates [22].

**Figure 1:**
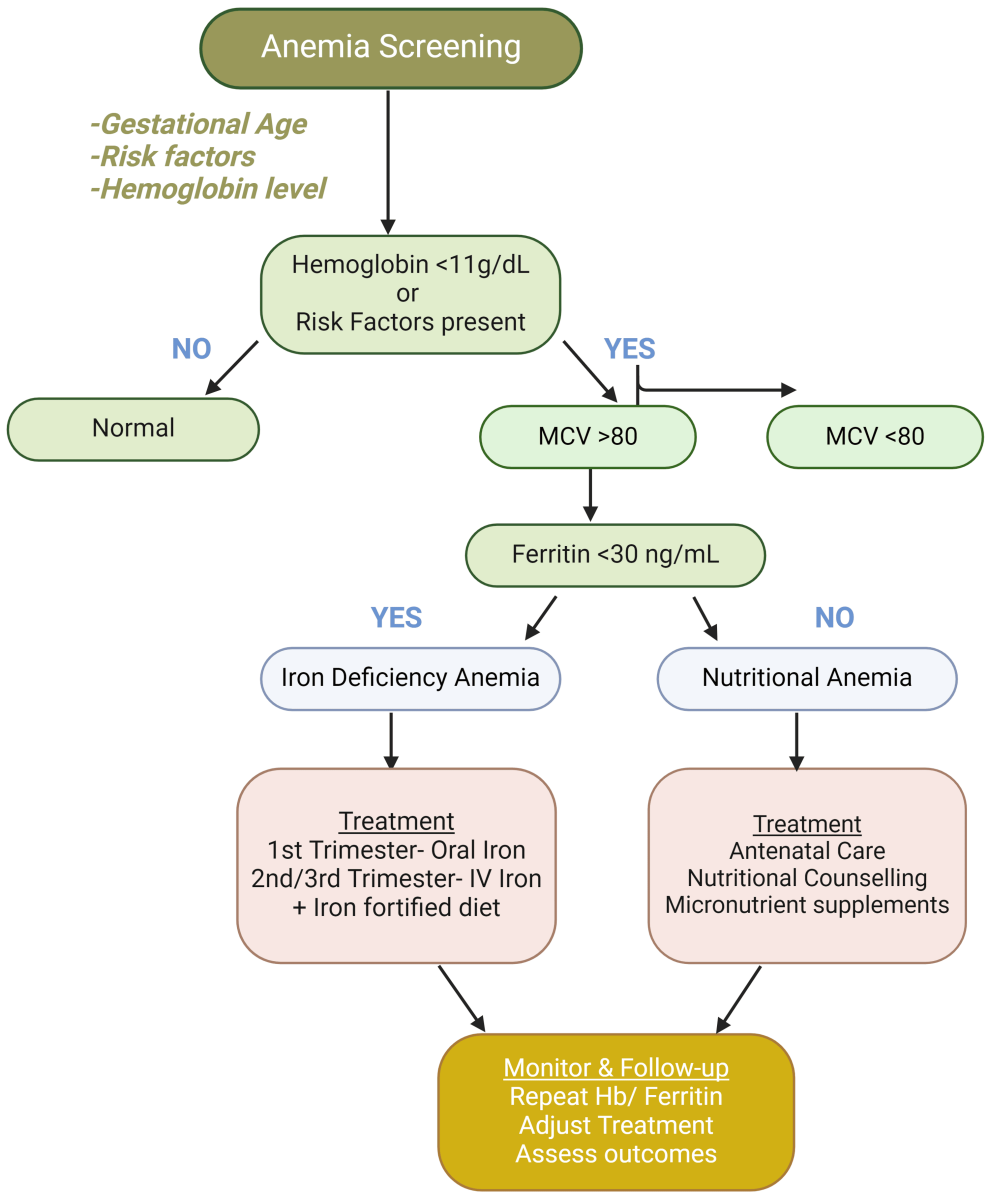
Algorithm for anemia screening, diagnosis, and treatment during pregnancy Original Image Created with Biorender

It is recommended that patients of childbearing age receive iron supplementation. Oral supplementation during the first trimester, in terms of dosage, is debatable and administration at 48-hour intervals has been shown to increase its absorption. The recommended daily intake of elemental iron during pregnancy is 27 mg, and during breastfeeding, it should be decreased to 9 mg [20]. Intravenous (IV) administration of iron in the second and third trimester is safe during pregnancy and has the advantage of being a single dose. The maximum dose depends on the IV formulation of iron: low molecular weight iron dextran (1,000 mg); iron isomaltoside (1,000 mg); iron sucrose (200-300 mg per dose, 1,000 mg total dose) [20]. The expected adverse effects are minimal in contrast to the consequences of iron deficiency. Adverse effects of oral administration entail mainly gastrointestinal effects, and IV administration includes anaphylactic reactions, headaches, and/or hypotension [20]. IV iron serves as the basis for improving maternal hemoglobin levels. Moreover, it decreases the chances of complications in the mother, including PPH and maternal death [16,20]. Strategic methods must be developed specific to various groups taking into account factors such as nutritional status, socio-economic considerations, and underlying health conditions. Additionally, the cost-effectiveness and efficacy of screening for IDA in obstetrics using blood tests like ferritin need further assessment [19]. IDA is diagnosed by a ferritin level less than 30 mg/dL (Figure 1) [16]. Although guidelines suggest hemoglobin assessment at regular intervals, particularly in high-risk pregnancies, there is not enough information on the therapeutic usefulness of ferritin testing. This warrants careful interpretation of existing recommendations.

The RANI project carried out in the Odisha state of India aimed to reduce anemia in pregnancy by implementing behavior modification treatments based on local norms. In women of reproductive age, particularly in prevalent regions, this has been reassuring in addressing anemia. Yet, randomized controlled trials need to be done to review the efficacy of these therapies [23].

Comprehensively, understanding the nature of anemia is complex during pregnancy. It requires regulated approaches which may include prenatal and antenatal screening, provision of supplements, and behavioral support to reduce its negative impact on maternal and fetal health. Enhancing current therapies and exploring unique strategies can successfully tackle the global challenge of IDA in pregnancy, hence more research needs to be undertaken.

Nutritional anemia

Nutrition during pregnancy is another global public health issue. Poor nutrition and a lack of essential macro and micronutrients in mothers during pregnancy have been linked to higher rates of illness and death in mothers. It is also responsible for hindering the child's long-term growth and brain development. There have been incremental advances over the last two decades, yet the maternal and child nutrition goals in low and middle-income countries have not been fully attained, with disruptions caused by the COVID-19 pandemic in addition [2].

Antenatal care (ANC) is the primary point of contact for pregnant women who need health and nutritional guidance. The “WHO Antenatal Care Guideline for Positive Pregnancy Experience” advocates offering iron-folic acid (IFA) supplements, nutrition counseling, and weight monitoring as standard prenatal care practices to expand the reach of nutrition interventions for pregnant women [24]. Multivariable analysis of a cross-sectional study conducted in 21 Bangladeshi states revealed that effective nutrition service delivery is linked to facilities having good logistical readiness, consultations by paramedics and local health care providers, health care providers' understanding of maternal nutrition, improved communication between health care providers and clients, and the use of ANC cards [25]. 

It is time to consider using multiple micronutrient (MMN) supplementation as the primary choice for routine prenatal treatment, rather than iron and folic acid alone, especially to reduce stillbirths, small for gestational age (SGA) births, and low birthweight babies. Individual micronutrients and vitamin supplements have been shown to improve certain outcomes, such as calcium which has been shown to lower the incidence of pre-eclampsia and eclampsia as well as vitamin A supplementation which has been shown to increase serum/plasma retinol levels in mothers [26]. However, vitamin toxicities should also be carefully monitored.

Sickle cell disease

Women with SCD and their newborns face greater health risks during pregnancy. Maternal mortality is two to three times greater than healthy pregnant women. The risk of pre-eclampsia, thromboembolism, acute pain crisis, and sepsis are also elevated. Moreover, babies also risk stunted growth, stillbirth, preterm delivery, and death during childbirth [3,4]. The risk comes from sickle cell-induced vascular degeneration, exacerbated by pregnancy-induced cardiovascular stress. Blood transfusions before and through pregnancy reduce risks but might cause adverse reactions or alloimmunization. A multidisciplinary care team should prescribe specialized sickle cell therapy, frequent monitoring for early identification, and tailored transfusion regimens to balance benefits and hazards. Even with rigorous therapy, SCD pregnancy is high-risk. More research is needed to identify risk factors and evaluate strategies to enhance mother-infant outcomes [4].

Sickle cell trait (AS), typically does not cause harm and is regarded as innocuous; however, pregnancy might increase the possibility of complications. One such consistent correlation seems to be observed with sickle cell trait causing high susceptibility to venous thromboembolism (VTE) [27]. A meta-analysis study looked at how sickle cell disease genotypes affected pregnancy outcomes. SS genotypes (Hemoglobin S inherited from both parents) were associated with a higher incidence of maternal hypertension, infections, pain crises, intrauterine growth restriction, and maternal and perinatal mortality. The SC genotype (inheriting a gene for Hemoglobin S and Hemoglobin C from either parent) increased the likelihood of cesarean delivery, pre-eclampsia, pain crises, low birth weight, and perinatal death. This study concluded that the SS genotype had the poorest pregnancy outcome and the highest risks. However, SC pregnancies are still riskier than healthy pregnancies. Other genotypes require further testing and studies. All SCD genotypes need specialized prenatal care and monitoring attributed to a high risk of complications [28].

Currently, the preferred screening tests for SCD are high-performance liquid chromatography, sickle solubility testing, isoelectric focusing, or hemoglobin electrophoresis [29]. Accurate diagnosis can be done by DNA analysis for α thalassemia in babies with SCD. Also, distinguishing between mild and severe β+ thalassemia variants is vital due to their distinct clinical trajectories. There is no evidence supporting the need for β globin haplotyping [30]. 

For the treatment of SCD, the use of hydroxyurea (a class II teratogen) has been studied and broadly speaking, it was concluded that its teratogenic effect is dose-dependent and, if the dose is 15-25 mg/kg, it could be used safely during pregnancy. More than 100 cases of its use during pregnancy have been published in the literature and there was no evidence of malformations [31]. Although there are documented cases of the use of hydroxyurea during pregnancy, there is a need for studies that offer convincing evidence of the safety of its use during pregnancy and lactation. 

The diligent monitoring of potential issues during pregnancy is of utmost importance to provide prompt intervention when necessary. Recommended interventions include initiating folic acid consumption before conception, receiving vaccinations for pneumonia and influenza, contemplating prophylactic blood transfusion, additional oxygen provision, and determining the optimal timing and method of delivery by the prevailing risks of complications [3].

Alongside further studies, it is necessary to implement social programs that ensure the effective prevention of IDA, nutritional anemia, and SCA. The aim is to reduce the incidence of complications of these diseases through community efforts and the work of raising awareness in society, especially in women of reproductive age. 

Multiple pregnancies outcomes

**Figure 2:**
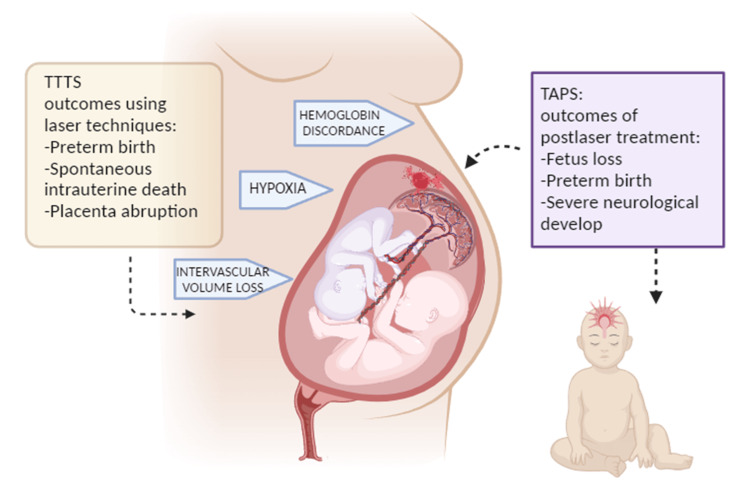
Overview of Outcomes of TTTS and TAPS, post-laser therapy TTTS: twin-to-twin transfusion syndrome; TAPS: twin anemia polycythemia syndrome Original Image Created with Biorender

Twin pregnancies with monochorionic placentation tend to have elevated risks of twin-to-twin transfusion syndrome and twin anemia polycythemia sequence. Twin-to-twin transfusion syndrome occurs when there is an alteration of amniotic fluid sacs between the donor and the recipient fetus [5]. The first line treatment for twin-to-twin transfusion syndrome is considered to be the fetoscopic laser technique [32]. The use of laser techniques could have negative neurological outcomes due to the ischemic brain areas seen in the donor and recipient twin, even though there was not enough insight into the neurodevelopment after birth since these lesions may vary depending on the time of the injury. Preterm birth and spontaneous intrauterine deaths were more likely to occur in twin pregnancies complicated by the twin-to-twin syndrome [33]. An alternative laser treatment used is the Salomon technique which compared to the fetoscopic laser photocoagulation has a better prognosis rate but still has shown a high incidence of preterm deliveries and a high risk of placenta abruption [34]. Twin anemia polycythemia syndrome is designated by the high discordance of hemoglobin in monochorionic twins [35]. Georgina et al. in their review stated that the incidence of post-laser twin anemia polycythemia sequence had a higher incidence of fetal loss and severe neurological development impaired compared to a spontaneous twin anemia polycythemia sequence; both factors have an increased risk of preterm birth (Figure 2) [36].

Morbidity and mortality are increased in triplet pregnancies. Curado et al. show us in their systematic review that chorionicity aims as a high factor for research since dichorionic triamniotic triplets generally have worse complications than in trichorionic triamniotic triplets, whereas hemodynamics changes are not seen in monochorionic pregnancies [37]. Risk factors of a triplet pregnancy are increased with preterm birth and intrauterine death when it is complicated with laser surgery for TTTS [38].

On a molecular level, there was a high incidence of angiotensin-converting enzyme 2, TMPRSS2, and Cathepsin B expression found on the marginal areas of placental tissue in twin pregnancies with anemia polycythemia sequence in women confirmed with SARS-CoV-2 which suggests hypoxic areas [39]. As a result of the decrease of the placental blood volume, the renin-angiotensin system is activated in the twin-to-twin transfusion syndrome where there remains a high concentration of blood [40]. The incidence of intrauterine death with intervention techniques like laser therapy and amnio-drainage in monochorionic monoamniotic twin pregnancy due to twin-to-twin syndrome was higher than having no treatment [41]. A helpful diagnostic tool for the antenatal detection of twin anemia polycythemia sequence is the delta middle cerebral artery peak systolic velocity (MCA-PSV) with Doppler measurements in monochorionic twin pregnancies [35,42].

ISUOG and NICE guidelines recommend performing ultrasound in uncomplicated twin pregnancies for routine monitoring every two weeks from week 16 of gestation, as an early way to detect TTTS and TAPS in monochorionic twin pregnancy, and every four weeks from week 20 of gestation in dichorionic twin pregnancy [43,44]. For complicated twin pregnancies with post-laser therapy and fetal growth, the restriction must be considered the utility of ultrasound using middle cerebral artery peak systolic velocity from week 16 for twin anemia polycythemia sequence [44]. Martinez-Portilla et al. sustain high evidence for the diagnosis of moderate-severe anemia using MCA-PSV above 1.5 MoM with fetuses that had no previous transfusions [42].

COVID-19 and pregnancy

Infection with SARS-CoV-2 disease can present with not exhibiting any symptoms of a multisystemic infection, making it hard to diagnose and provide an accurate treatment. The most common results of laboratory changes during pregnancy confirmed with COVID-19 were C reactive protein, lymphopenia, leukocytosis, and thrombocytopenia. These parameters may differ from mild to moderate infection, and in severe cases, alterations of D-dimer and elevated transaminase [6]. Thromboembolic and hemostatic outcomes are increased in pregnant women affected by COVID-19. The accuracy of these parameters is not reliable since they can also be elevated in other pathologies within pregnancy like HELLP syndrome, pneumonia, pulmonary embolism (PE), and preeclampsia, among others [7,8].

Positive outcomes are strongly seen in the third trimester of pregnancy from asymptomatic and mild cases [45]. Pregnant women with anemia associated with the severity of positive COVID-19 had a high risk of preterm birth, increased risk of ventilation, and fetal death [7,46]. Severe cases of COVID-19 during pregnancy should be managed with a multidisciplinary team. ISUOG suggests fetal monitoring after 26 to 28 weeks of pregnancy, fetal growth assessment, and amniotic volume analysis with ultrasound [47]. Prognosis is not completely defined since COVID-19 can affect every pregnant woman and develop in a different form with or without pathologies, complications may differ from mild to severe cases, and so is its treatment. Further detailed investigation needs to be deepened. 

Malaria and pregnancy

Malaria infection during pregnancy presents unique and challenging health concerns due to the complexity of maternal physiology, and the interaction between the maternal immune system, placental biology, and plasmodium parasite pathogenicity. The presence of malaria gives rise to a spectrum of complications for the mother and the developing fetus, such as anemia, miscarriage, stillbirth, low birth weight, preterm delivery, and even maternal death [9]. In 2019, there were 229 million cases globally, with Africa accounting for 94% of them [48]. Approximately 125 million expectant mothers live in regions where they are exposed to a potential risk of acquiring malaria. Malaria is caused by the Plasmodium parasite. Adverse maternal and fetal outcomes are most associated with *Plasmodium falciparum* and *Plasmodium vivax*. *P. falciparum* is the most common species of malaria in sub-Saharan Africa, and it is considered the most lethal [49]. Malaria is spread through the bite of female Anopheles mosquitoes, hence variables that influence anopheles abundance consequently impact malaria incidence. In 1940 malaria was eliminated from the southern United States through extensive dichlorodiphenyltrichloroethane (DDT) spraying to exterminate anopheles mosquitoes. In recent decades, progress has been made particularly in Europe, and parts of Central and South America in reducing the prevalence of malaria. Nevertheless, women continue to face a substantial risk of malaria during pregnancy. Over 50% of women in high transmission areas have *P. falciparum* detected in their blood upon presentation for antenatal care. According to the World Health Organization, nearly 25 million pregnant women face the risk of malaria infection during pregnancy. These infections result in 200,000 neonatal and 10,000 maternal deaths annually. These numbers account for over 90% of global malaria mortality rates concentrated in that region [9]. 

In general, pregnant women face an increased likelihood of contracting malaria and experiencing symptomatic disease, as compared to non-pregnant women with mortality rates reaching 50% in endemic areas [9]. However, various factors can influence disease risk and severity such as immunity status, age, nutrition, parity, gestational age, and the region of residence. Adolescents, malnourished, and women with HIV, have shown high susceptibility to malaria in certain settings. Studies have also shown that malaria occurrence is highest during the first pregnancy, reaching its peak in the second trimester, and subsequently declining as the pregnancy progresses toward term [50]. Additionally, recent studies involving 300 women giving birth in Ghana revealed a high incidence of anemia, malaria infection, and a high level of placental infection burden in primigravida as compared to multigravida [9]. Area of residence is also an important factor in disease complication and severity. In regions with low malaria transmission rates, maternal illness tends to be more severe, because of the lack of pre-existing/acquired immunity among pregnant women, putting them at higher risk of developing cerebral malaria and pulmonary edema compared to non-pregnant women. Moreover, women in low transmission regions are at increased risk of miscarriages and stillbirths in contrast to women who live in areas with high malaria transmission rates. However, in these areas, both primigravida and multigravida are at equal risk of malaria infection [50].

It is suggested that the majority of pregnancy-related complications arise from the sequestration of infected erythrocytes in the placenta and the immunocompromised state during pregnancy [9]. P. falciparum has been frequently associated with the manifestation of more severe symptoms, leading to a higher mortality rate compared to other malaria species [49]. Pathogenesis of *P. falciparum* is marked by the adhesion of infected erythrocytes to the vascular endothelium of the placenta. The parasite expresses a unique protein on the membrane of the infected erythrocytes called VAR2CSA. This protein aids the adherence between the infected erythrocytes and chondroitin-sulfate A on the endothelium of placental blood vessels and parasite accumulation in the intervillous spaces. The presence of *P. falciparum* and its disposal product (hemozoin) in the intervillous spaces triggers monocytes and macrophage infiltration, and subsequently inflammation of the placenta. Placental inflammation leads to alteration in both villous architecture and placental angiogenesis, resulting in disruption of uteroplacental blood flow. These structural and functional changes in the placenta have been confirmed using longitudinal Doppler data which showed that malaria during the first half of pregnancy is associated with changes in the umbilical artery blood flow [46].

Impact of pregnancy-associated malaria on the mother and the fetus

**Figure 3:**
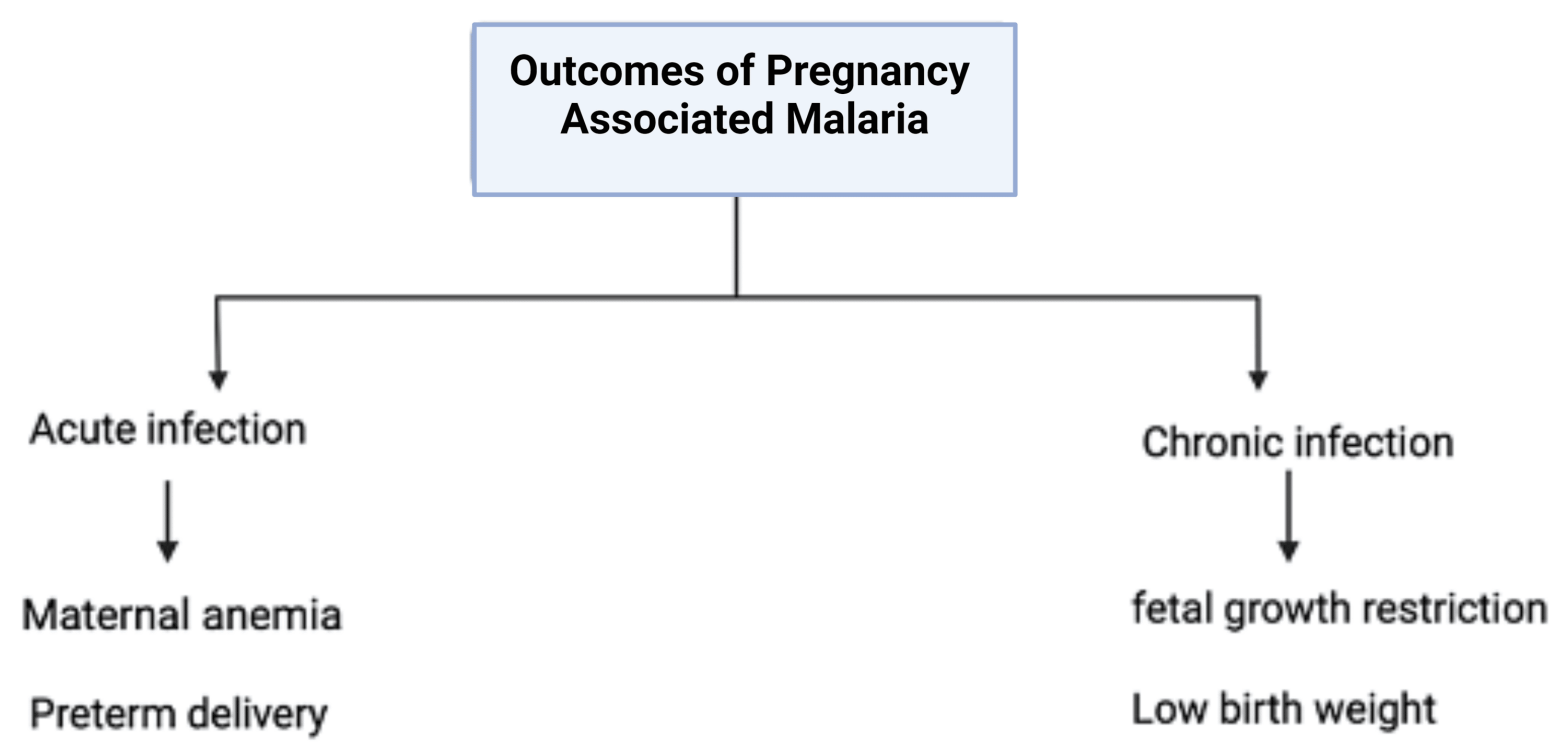
Flowchart representing the outcomes of malaria in pregnancy Original Image Created with Biorender

Acute malaria infection with a high parasite load has been linked to preterm delivery, however, chronic infections are associated more so with fetal growth restriction and low birth weight (Figure 3) [51]. Chronic P. falciparum infection is associated with more incidences of placental inflammation than acute infection due to the deposition of significant amounts of hemozoin, leading to more inflammatory cell infiltration and fibrin deposition. Placental inflammation is due to hemozoin deposition and inflammatory cell infiltration, which leads to changes in placental angiogenesis, and disruption of umbilical blood flow compared to acute infection [51]. *P. vivax*, in contrast to *P. falciparum*, is not associated with placental sequestration. Placental infection with *P. vivax* does not display any pathological features, indicating that *P. vivax* induces low birth weight through systemic rather than local changes in the placenta and the umbilical cord [50]. Chronic malaria infection is also associated with maternal anemia. In Africa, malaria is estimated to account for 25% of cases of maternal anemia, due to erythrocyte sequestration and folic acid deficiency. Severe anemia in pregnancy increases the likelihood of complications such as intrapartum hemorrhage, congestive heart failure, and fetal demise [9]. Chronic malaria is also associated with gestational hyperinsulinemia and hypoglycemia. It is believed that erythrocyte sequestration stimulates insulin production by beta cells in the pancreas. Additionally, malaria and HELLP (hemolysis, elevated liver enzymes, low platelet counts) syndrome overlap in the clinical and laboratory findings, as erythrocyte sequestration can also happen in small blood vessels of the brain and the kidneys, resulting in cerebral malaria, renal failure, and thrombocytopenia. Misdiagnosis can result in delayed appropriate management and increased fetal and maternal morbidity and mortality [9]. The immune state of pregnant women is another factor that can affect the outcomes of malaria infection, pregnant women who have HIV infection are at increased risk for malaria infection and its associated complications. About 50 million women each year conceive in areas with high malaria prevalence, among these women it is estimated that around one million pregnant women are co-infected with both malaria and HIV. HIV is associated with high levels of parasitemia, resulting in a higher incidence of maternal anemia and low birth weight. Additionally, a high HIV viral load raises the likelihood of malaria infection and the severity of the complications [9]. Congenital malaria is an uncommon complication of maternal malaria, with an incidence rate of 0.3% in women with acquired immunity and 7.4% in non-immune women. It is characterized by the presence of malaria parasites in the peripheral blood of the newborn within the first seven days of life [51,52].

Diagnosis of malaria during pregnancy

There are two diagnostic tools for pregnancy malaria available for clinical settings: blood smear microscopy, which is considered the gold standard due to its extensive clinical use, and rapid diagnostic tests (RDT), which are capable of identifying soluble plasmodium antigens in the blood. Specifically, RDT-HRP2 sensitivity exceeds 90% when compared to peripheral blood smear for diagnosing placental malaria, and ranges from 80% to 95% when compared to placental blood smear. However, their specificity varies between 61% and 94%. Nevertheless, the sensitivity of RDT-HRP2 using peripheral blood samples is notably lower in contrast to PCR detection of parasite nucleic acid in placental or peripheral blood [53]. Polymerase chain reaction (PCR), including quantitative PCR, is another diagnostic tool that can be used for malaria diagnosis. It is highly sensitive and can detect even very low-density malaria infection. However, PCR requires specialized laboratories with trained personnel, and it is relatively time-consuming, making it unsuitable for point-of-care applications and only used in laboratory settings [54].

Placental histology is not commonly used for diagnosing malaria during pregnancy, but it plays a crucial role in research studies and clinical trials focusing on pregnancy-related malaria. It is considered the gold standard because it can detect parasites sequestered in the placenta even when they are not found in the bloodstream [53]. Active malaria infections, especially in first-time mothers (primigravidas), are associated with low birth weight and inflammation with monocyte infiltration in the placenta. On the other hand, past infections show features like fibrin deposition and the presence of malaria pigment (hemozoin). Hemozoin can persist in the body for months after treatment and is linked to cumulative exposure to malaria [53,54].

Thrombophilia in pregnancy

Thrombophilia is a medical condition marked by a greater likelihood of developing blood clots. It can be caused by various factors, including genetic mutations, acquired conditions, or a combination of both. It results in arterial or venous thrombosis due to changes in one or more of the elements involved in hemostasis, such as plasma proteins, coagulation factors, vascular surface, or blood flow [55]. Thrombophilia has been linked to a range of significant complications for the mother and the fetus during pregnancy; such as recurrent pregnancy loss, late miscarriages, stillbirth, intrauterine growth restriction, preeclampsia, and HELLP syndrome (hemolysis, elevated liver enzymes, low platelet counts) [10].

In general, the risk of thrombosis is higher during pregnancy, due to the significant hormonal shifts and reduced mobility. The risk varies depending on the gestational age, mode of delivery, and the associated comorbidities if present. A higher risk of thrombosis is observed during the third trimester as compared to the first trimester. This risk increases during the postpartum period. It is also observed that cesarean section delivery carries a higher risk of thrombosis compared to normal delivery. Additionally, maternal diabetes, obesity, heart disease, and smoking are also associated with a high risk of thrombotic events [56].

Thrombophilia can be classified according to etiology into hereditary thrombophilia and acquired thrombophilia. Hereditary thrombophilia can be subdivided into two categories: 1) deficiency in coagulation inhibitors, like antithrombin III deficiency (AT III), protein C, and protein S deficiency and 2) elevation in coagulation factors, such as dysfibrinogenemia, increased levels of factors VIII, XI, IX, factor V Leiden, activated protein C resistance (APC), and prothrombin gene mutation. The most common types of hereditary thrombophilia are factor V Leiden and prothrombin gene mutation, which together account for approximately 70% of diagnosed cases with hereditary thrombophilia. Protein S and C deficiency and antithrombin III deficiency are more severe but less frequent [55]. Acquired thrombophilia on the other hand, is due to acquired disorders of hemostasis, these acquired conditions may promote a prothrombotic state by increasing levels of procoagulant factors, reducing levels of anticoagulants, or inducing inflammation in blood vessels [55,57]. The primary major disorders associated with thrombophilia are hyperhomocysteinemia and antiphospholipid antibody syndrome [55].

Antiphospholipid syndrome is an autoimmune disorder characterized by the presence of antiphospholipid antibodies in the blood, such as lupus anticoagulant, anti-β-2-glycoprotein 1, and/or anticardiolipin antibodies. These antibodies cause blood clotting by inducing inflammation in blood vessels. They target phospholipids, a type of fat found in cell membranes of different organs, including the placenta. This process leads to the production of tissue factors and proinflammatory cytokines by placental tissues. This further activates the complement system resulting in more recruitment of inflammatory cells [57].

Hyperhomocysteinemia is due to elevation of the homocysteine blood level of more than 5 mmol/L on an empty stomach or more than 51 mmol/L after administration of methionine. Homocysteine is produced as a byproduct of methionine metabolism, which is an essential dietary amino acid. In normal pregnancies, the level of homocysteine tends to decrease. This decrease is attributed to hemodilution resulting from the expansion of blood volume and increased glomerular filtration rate during pregnancy. Furthermore, a fraction of homocysteine is absorbed by the fetus during gestation. Homocysteine levels decrease in early pregnancy, reach their nadir in the second trimester, and then gradually rise during late pregnancy to levels seen in early pregnancy [58].

“The normal values of Hct during pregnancy are as follows: 3.9-7.3 mmol/L before 16 gestational weeks, 3.5-5.3 mmol/L between 20 and 24 gestational weeks, and 3.3-7.5 mmol/L after 36 gestational weeks” [58]. Several factors can contribute to elevated homocysteine (Hct) levels, including genetic defects such as methylenetetrahydrofolate reductase (MTHFR) gene mutation, deficiencies in vitamin B6, B12, and folic acid, as well as hypothyroidism, certain medications, renal dysfunction, and aging. Elevated homocysteine levels can lead to thrombosis due to endothelial cell injury; they generate superoxide and hydrogen peroxide free radicals which induce endothelial cell apoptosis. Additionally, high homocysteine levels reduce the release of nitric oxide by endothelial cells and promote platelet aggregation and thrombus formation [58]. 

The risks of thrombophilia during pregnancy vary according to the etiology, antithrombin III deficiency and protein S and C deficiency are considered high-risk factors for thrombophilia during pregnancy. While activated protein C resistance, factor V Leiden, and prothrombin gene mutation carry moderate risk, lower still is hyperhomocysteinemia [55].

Thrombophilia and adverse pregnancy outcomes

Both acquired and hereditary thrombophilia account for over 50% of diagnosed thrombotic events during pregnancy, delivery, and the postpartum period. They can lead to significant complications due to the disruption of the normal placental blood flow and the normal evolution of the placenta, resulting in risks for both the mother and the fetus in terms of morbidity and mortality. These complications include venue and arterial thromboembolism, pre-eclampsia, and HELLP syndrome [59]. Recurrent miscarriages, first-trimester abortion, mid-trimester abortion, preeclampsia, fetal growth restriction, placental abruption, or intrauterine death [55]. In the Western world, PE stands as a primary cause of maternal mortality, while VTE contributes significantly to maternal morbidity. Studies report VTE incidence rates ranging from 0.6 to 1.3 episodes per 1,000 deliveries, representing a five to 10-fold increase in risk compared to non-pregnant women of similar age. A meta-analysis study demonstrated that two-thirds of deep venous thrombosis occurs before delivery, with evenly distributed risk across the three trimesters, whereas 43% to 60% of pregnancy-related PE occurs in the four to six weeks postpartum. In general, the risk of PE and deep venous thrombosis is significantly higher post-delivery compared to the antenatal period [60].

Placental abnormalities linked with thrombophilia and pregnancy complication

In one study, placental pathology in cases of early-onset preeclampsia and fetal growth restriction showed no significant differences irrespective of the presence of thrombophilia, although higher rates of placental abnormalities were detected [61]. Another study involved 13 women with thrombotic lesions of the placenta, all participants experienced obstetric complications such as preterm labor, preeclampsia, intrauterine growth restriction, or stillbirth. The study identified inherited thrombophilia in 10 of the 13 women, 77%. Specifically, seven were heterozygous for the FV Leiden mutation, and three had protein S deficiency. The most observed placental lesions included fetal stem vessel thrombosis, infarcts, spiral artery thrombosis, hypoplasia, and peri-villous fibrin deposition [62]. 

The increased prevalence of placental lesions observed in both thrombophilia and non-thrombophilia women experiencing severe complications in these studies presents a challenge in differentiating between the two groups or suggests a potential undiscovered aspect of thrombophilia. It is essential to acknowledge that these studies evaluated various clinical conditions beyond just severe preeclampsia [63].

Screening for thrombophilia during pregnancy

The utility of thrombophilia screening is subject to debate. However, it is worth noting that laboratories are rapidly developing various sets of tests for diagnosing thrombophilia. The first recorded screening for hereditary thrombophilia was in 1965. This was found in a family diagnosed with serine protease inhibitor deficiency. Subsequently, several abnormalities have been associated with hereditary thrombophilia, prompting laboratories to develop a comprehensive array of tests to identify patients with these abnormalities [55].

**Figure 4:**
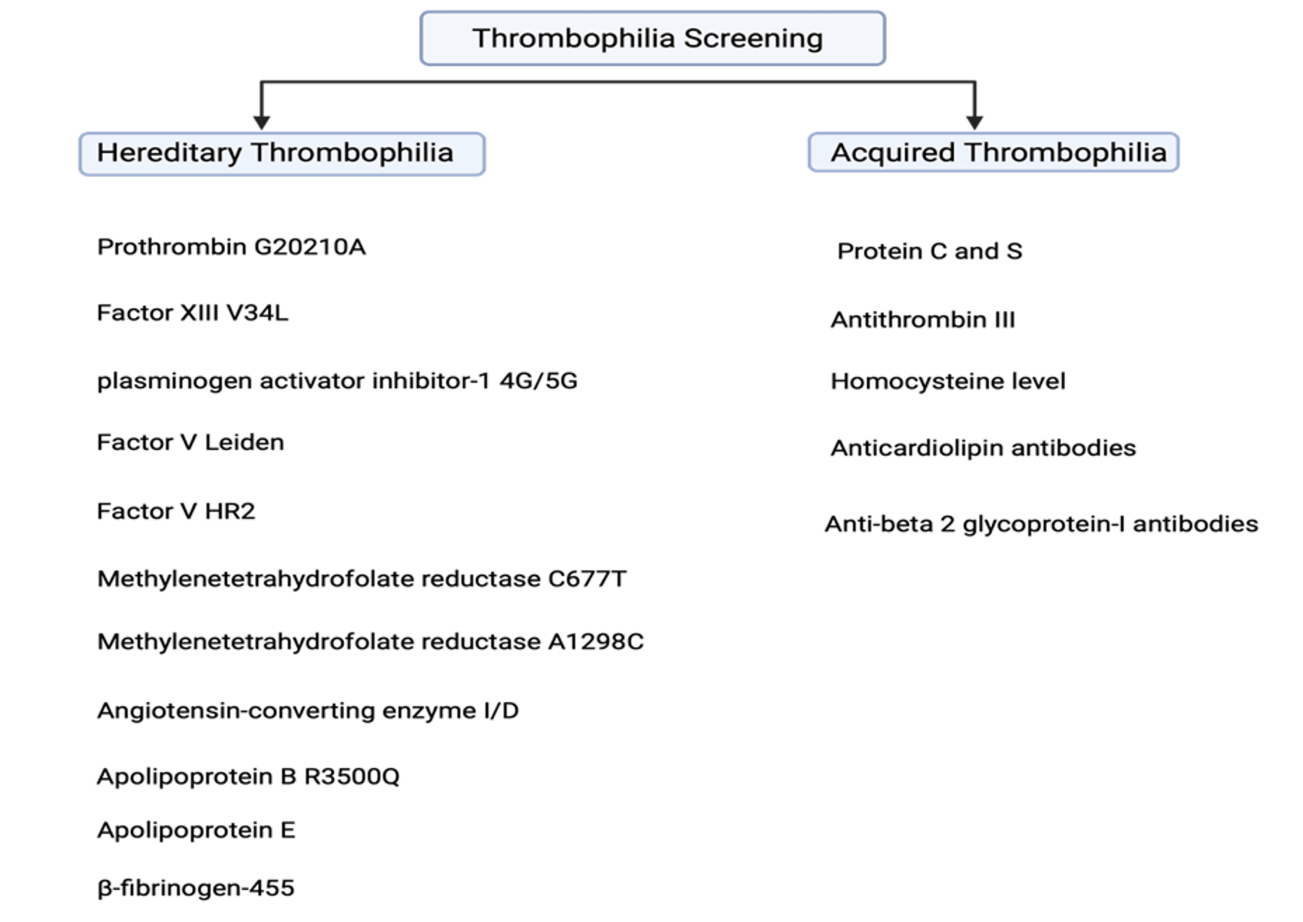
Thrombophilia screening

Screening for thrombophilia involves a diverse range of coagulation and genetic tests. This includes array of tests, including genetic parameters such as prothrombin G20210A, factor V Leiden (FVL), factor XIII V34L, factor V HR2, plasminogen activator inhibitor-1 4G/5G (PAI-1), MTHFR C677T, MTHFR A1298C, β-fibrinogen-455, apolipoprotein E (Apo E), angiotensin-converting enzyme I/D, apolipoprotein B R3500Q. Additionally, measurements of protein C and S, antithrombin III, and analysis of homocysteine levels are conducted as part of the screening process. Screening for thrombophilia also includes tests to identify lupus anticoagulants, anticardiolipin antibodies, and anti-beta 2 glycoprotein-I antibodies to confirm or rule out the presence of antiphospholipid syndrome (Figure 4) [55,56].

Genetic testing for thrombophilia is expensive and requires expertise for accurate interpretations, hence determining the appropriate timing and candidate population for these specific tests is crucial. As such, specific tests for thrombophilia should not be conducted during a thrombotic event, as the result may be affected by various factors. Additionally, the diagnosis of hereditary thrombophilia has no impact on the primary management of thromboembolism. It is important to consider the medications taken during testing, as certain drugs like low molecular weight heparin (LMWH) can affect antithrombin levels. While screening for acquired thrombophilia conditions is recommended in cases of thrombosis, testing for hereditary conditions may not always be beneficial [55].

Clinical evaluation for thrombophilia is also considered part of the screening process. Clinical evaluation is recommended for patients with a history of VTE or women who have specific complications of pregnancy such as preeclampsia, recurrent miscarriages, fetal growth restriction, placental abruption, first-trimester abortion, mid-trimester abortion, and intrauterine deaths. The evaluation includes a review of family and personal medical history, including past thrombotic events and associated risk factors. A comprehensive physical examination, focusing on skin, lymphatic, peripheral arterial and venous, cardiorespiratory, abdominal, urinary, and neurological systems, is also advised [55]. 

Management of hereditary thrombophilia during pregnancy according to the ACOG and RCOG

The American College of Chest Physicians (ACCP) guidance suggests LMWH prophylaxis for two groups of women: those without a family history of VTE but with homozygosity for FVL or prothrombin gene mutations, and women with a family history of VTE along with any other inherited thrombophilia. However, LMWH prophylaxis is not recommended for women with inherited thrombophilia in the absence of previous pregnancy complications due to insufficient evidence of improved pregnancy outcomes. Aspirin is recommended for those at high risk of pre-eclampsia, regardless of thrombophilia history, aligning with ACOG recommendations and supported by robust evidence. [64].

The Royal College of Obstetricians and Gynecologists (RCOG) guidelines advise considering LMWH prophylaxis for women with antithrombin, Protein C, or Protein S deficiency, even in the absence of a family or personal history of VTE, which contrasts with the ACCP guidance. They also suggest considering antenatal LMWH prophylaxis for asymptomatic individuals with homozygosity for FVL and prothrombin mutation. The RCOG guidelines further stratify risk based on the accumulation of risk factors, recommending LMWH prophylaxis if heterozygosity for FVL or prothrombin gene mutation is present along with two or three other risk factors or compound heterozygosity. Unlike the ACCP guidance, the RCOG guidelines apply risk stratification to dosing differences, recommending 50-100% treatment dose antenatally and for six weeks postnatally for women with antithrombin deficiency and previous VTE [64].

Anticoagulant therapy during delivery

To prevent the adverse effects of anticoagulants during delivery, especially with neuraxial anesthesia, it is recommended to discontinue low molecular weight heparin (LMWH) or unfractionated heparin (UFH) 24 to 36 hours before elective induction of labor or cesarean section. Neuraxial anesthesia is contraindicated in women who are on anticoagulants if they go into spontaneous labor. It is essential to monitor aPTT in women receiving subcutaneous UFH, protamine sulfate may be required when aPTT is significantly prolonged to minimize the risk of bleeding. It is also important to do an Anti-Xa assay in women who are on LMWH or unfractionated heparin.

If bleeding occurs in women who are on LMWH, protamine sulfate can be used for partial neutralization. While recombinant activated factor VII concentrate has been successfully employed to reverse LMWH-induced bleeding in nonpregnant patients with hypercoagulable states, its use in pregnancy is limited due to concerns about thrombogenicity. Hence, this intervention should be reserved for major bleeding cases unresponsive to conventional therapy [60].

## Conclusions

Maternal and fetal morbidity and mortality are impacted greatly by maternal hematological disorders. These disorders can affect both the mother and the fetus. Clotting disorders, anemia, and other hematological disorders, along with blood infections, like malaria, increase the health risks. These risks include preterm labor, intrauterine growth restrictions, low birth weight, maternal hypertension, venous thrombosis, and miscarriage. Optimizing outcomes and reducing the impact of these health risks can be accomplished through a multidisciplinary approach. Involving obstetricians, pediatricians, and hematology specialists can ensure diagnosis. Moreover, genetic screening for hereditary blood disorders can be utilized to develop personalized treatment plans essential for reducing risks and promoting positive pregnancy outcomes.
